# Clinical and Linguistic Correlates of Functional Communication Abilities After Stroke: A Longitudinal Study

**DOI:** 10.3390/brainsci15101027

**Published:** 2025-09-23

**Authors:** Pasquale Moretta, Laura Marcuccio, Nicola Davide Cavallo, Roberta Galetta, Rosanna Falcone, Vittorio Masiello, Gerardo Cavaliere, Carlo Miccio, Emilia Picciola, Ernesto Losavio, Simona Spaccavento

**Affiliations:** 1 Istituti Clinici Scientifici Maugeri IRCCS, Department of Neurorehabilitation, Institute of Telese Terme Via Bagni Vecchi 1, 82037 Telese Terme, Italy; laura.marcuccio@icsmaugeri.it (L.M.); nicola.cavallo@icsmaugeri.it (N.D.C.); vittorio.masiello@icsmaugeri.it (V.M.); gerardo.cavaliere@icsmaugeri.it (G.C.); carlo.miccio@icsmaugeri.it (C.M.); 2 Istituti Clinici Scientifici Maugeri IRCCS, Department of Neurorehabilitation, Institute of Bari, Via Generale Bellomo 73/75, 70124 Bari, Italy; roberta.galetta@icsmaugeri.it (R.G.); rosanna.falcone@icsmaugeri.it (R.F.); emilia.picciola@icsmaugeri.it (E.P.); ernesto.losavio@icsmaugeri.it (E.L.); simona.spaccavento@icsmaugeri.it (S.S.)

**Keywords:** aphasia, stroke, functional communication, rehabilitation, comprehension, language recovery

## Abstract

**Background:** Aphasia, a common consequence of left-hemisphere stroke, significantly impairs communication and daily functioning. Various studies have explored language recovery but only few have focused on the predictors of recovery of functional communication in patients with stroke. **Objective:** To identify clinical and linguistic factors associated with functional communication outcomes in patients with post-stroke aphasia. **Methods:** We enrolled 61 patients with aphasia due to left-hemispheric stroke, admitted to post-acute neurorehabilitation centers. Patients underwent neuropsychological, functional, and language assessments at admission (T0) and discharge (T1). Language abilities were evaluated with the Brief Exam of Language—II (BEL-II), and functional communication was measured through caregiver-rated I-CETI scores. Depression, basic (ADL) and instrumental (IADL) activities of daily living were also assessed. Correlations and regression models were used to examine predictors of functional communication recovery (ΔCETI). **Results:** Significant improvements were observed in all language domains, functional independence, and mood symptoms from T0 to T1 (*p* < 0.003). Regression analysis showed that demographic and general clinical variables (e.g., age, etiology, dysphagia) were not significant predictors of ΔCETI. However, ADL score, comprehension skills (Token test and comprehension sub-score of BEL-II) were significantly associated with functional communication recovery (β = 0.51, β = 0.68 and β = 0.75, respectively; *p* < 0.05). **Conclusions:** Functional communication recovery in post-stroke aphasia is strongly associated with initial comprehension abilities and functional autonomy in basic life activities, rather than demographic or general clinical variables. These findings highlight the need for targeted interventions aimed at improving receptive language and the importance of including ecologically valid communication assessments in post-stroke rehabilitation protocols.

## 1. Introduction

Aphasia is an acquired language disorder that occurs following brain injury, such as ischemic or hemorrhagic stroke, traumatic brain injury, or brain tumors. Approximately one-third of post-stroke patients experience aphasia, most often resulting from a lesion in the left hemisphere [1]. This language impairment affects verbal expression, auditory comprehension, reading, and writing, leading to significant limitations in communication activities. Although linguistic skills and functional communication abilities are distinct, they are strongly linked [2].

Language impairment significantly affects a patient’s functional outcomes and disrupts social, familial, and occupational relationships. Additionally, this communication deficit often has emotional and psychological consequences for both patients and caregivers, negatively impacting quality of life. Patients with aphasia frequently experience high levels of depression, social exclusion, loss of social contacts, and reduced quality of life [3,4].

Unfortunately, even after targeted speech therapy, the recovery of language skills sometime could not determine improvements in daily life communication because deficits in communicative interactions are not necessarily related to severity of the linguistic problems and are based on different abilities and affect the so called “functional communication” [5,6,7].

Functional communication is defined as “the ability to receive and convey messages effectively and independently, regardless of the mode of communication, in a natural context” [8]. It involves not only the accurate production and comprehension of spoken and written language, but also the use of gestures, facial expressions, and contextual cues to convey meaning, make requests, express needs, engage in social interaction, and respond to communication partners [8].

In individuals with aphasia, functional communication is often significantly impaired due to disruptions in the neural networks responsible for language processing. These deficits may affect various aspects of communication, such as finding words (anomia), constructing grammatically correct sentences (agrammatism), understanding spoken or written language, or producing fluent speech. As a result, people with aphasia may struggle to initiate or maintain conversations, respond appropriately, or communicate basic needs, which greatly impacts their independence, quality of life, and social participation [8,9].

Communication outcomes in individuals with aphasia depend not only on language deficits but also on impairments in other cognitive domains, such as attention, memory, and executive functioning. Significant correlations have been found between attention deficits and language and communication status in people with aphasia [9].

Despite this clinical relevance, few studies have systematically investigated the clinical and linguistic factors influencing the recovery of functional communication in post-stroke aphasic patients [10]. Recently, Jacobs et al. [11] explored the relationship between social and demographic factors and language deficits in people with aphasia, finding that age, time post-onset, race, and family size were associated with language performance.

Given the significant impact on functional autonomy, outcome communication measures are essential in the clinical evaluation of patients with aphasia, also serving to tailor language and cognitive rehabilitation programs.

Several measures have been developed to assess the functional autonomy of people with aphasia in everyday activities [12]. Examples include the American Speech-Language-Hearing Association Functional Assessment of Communication Skills (ASHA-FACS) [13,14,15], the Functional Outcome Questionnaire for Aphasia (FOQ-A) [16,17], and the Communicative Effectiveness Index (CETI) [18,19], all of which are specifically designed for patients with aphasia.

Moreover, only a limited number of studies have used specific tools designed to evaluate functional communication, such as the CETI [18,19], while most have relied on more general motor or quality of life assessments (e.g., Functional Independence Measure or Barthel Index) [3,20].

Recently, validated tools for assessing functional communication in everyday contexts have become available in Italian. These include the FOQ-A [16,17] and the Italian version of the CETI (I-CETI) [18,19], both providing ecologically valid assessments of communication skills. Both measures demonstrate good psychometric properties and are useful in evaluating the functional outcomes of aphasia rehabilitation in Italian-speaking patients.

Previous research has highlighted the impact of sociodemographic factors, such as education, and clinical characteristics, such as stroke severity, on communication outcomes [15,16]. Kim et al. (2016) [21] found that patients with both aphasia and dysarthria experience more severe strokes, greater cognitive deficits, and worse quality of life compared to those with aphasia alone. However, these patients still showed significant gains following rehabilitation, although no specific communication outcome measures were used in this study. Fernandes et al. (2022) [10], using the ASHA-FACS scale [13,14,15], found that functional independence at discharge was a significant predictor of communication recovery, demonstrating that functional communication is related to autonomy in daily living activities, as measured by the modified Barthel Index.

These findings emphasize the need for a multidimensional assessment of communication outcomes that considers both linguistic and non-linguistic factors. In line with this, Schumacher et al. [22] recommended using multiple measures to capture the complex nature of functional communication, which may be influenced by phonological level of language, non-verbal, and cognitive abilities.

Within this context, the present study aims to evaluate demographic, clinical and linguistic correlates of functional communication outcomes in patients with post-stroke aphasia, using standardized assessments of both language and functional abilities.

## 2. Materials and Methods

### 2.1. Participants

We screened a consecutive series of patients with aphasia due to left-hemisphere stroke admitted to post-acute rehabilitation units of Maugeri Clinical Institutes located in Telese Terme and Bari, from September 2018 to April 2019. All patients were selected for the study on the basis of the following inclusion and exclusion criteria.

Inclusion criteria: (a) age ranging 18 to 75 years; (b) at least 2 weeks from disease onset; (c) Italian-speaking patients; (d) diagnosis of aphasia according to clinical criteria (i.e., errors in verbal output including word-finding problems, impairments in understanding speech, and difficulty in reading and/or writing); (e) presence of Italian-speaking caregiver.

Exclusion criteria: (a) presence of degenerative disease or traumatic brain injury; (b) presence of behavioral disorders; (c) presence of speech disorders due to psychiatric disturbances.

### 2.2. Procedures

The study protocol included two assessments: one at study entry (T0), within two weeks of admission to the Rehabilitation Unit, and another at discharge (T1), following two months of intensive speech therapy (an average of 200 min at week, 5 days/week based on a neuropsychological approach) and neuromotor rehabilitation (an average of 330 min at week, 6 days/week based on Bobath concept) [23,24]. At both T0 and T1, an expert neuropsychologist assessed patients’ language domains and functional abilities, while caregivers evaluated their functional communication skills to provide a measure independent of the clinicians’ assessments by means of standardized questionnaire. During the assessment sessions, the following variables were collected: (i) clinical and demographical information such as age, sex, time post-injury, etiology (ischemic vs. hemorrhagic), presence of tracheotomy, presence of dysphagia (presence of dysphagia was evaluated according to clinical criteria and confirmed by a DOSS score < 7) at T0; (ii) at discharge, about two months after admission (T1), we re-evaluated clinical variables and language abilities.

### 2.3. Language and Functional Assessment

To assess language impairment, we used the Brief Exam of Language—II (Esame del Linguaggio Breve, BEL-II) [25], a standardized assessment battery for evaluating residual language abilities in aphasic patients. It includes six subtests that examine oral and written spontaneous production, naming, comprehension, and transcoding tasks (repetition, reading aloud, and writing under dictation). All subtest scores are expressed as percentages of correct responses (range: 0–100), with higher scores indicating better language abilities. The full battery takes approximately 30 min to administer and 15–20 min to score. The total score, representing the mean of the subtest scores, ranges from 0 to 100.

To assess receptive language abilities, particularly comprehension of spoken commands, we used the Token Test [26]. This is a widely used neuropsychological tool in clinical settings involving patients with stroke, brain injury, or neurodegenerative diseases. The test involves 20 tokens that vary by shape (circle/square), size (large/small), and color (e.g., red, green, black, white, yellow). It comprises five parts of increasing linguistic complexity: (i) simple commands (e.g., “Touch the red circle”); (ii) complex commands with multiple descriptors; (iii) commands involving more than one token (e.g., “Put the red circle on the green square”); and (iv) commands with embedded clauses, and long, syntactically complex commands.

The test consists of 62 items, and the total score ranges from 0 to 62. Errors can be categorized (e.g., omissions, substitutions, sequencing errors), allowing for more detailed analysis of comprehension deficits.

Functional communication abilities were assessed using the Italian version of the Communicative Effectiveness Index (I-CETI) [19], a validated and standardized questionnaire with 16 items measuring verbal and non-verbal communication skills in patients with aphasia. Respondents rate each item on a visual analog scale (0–100 mm), with 0 indicating “not at all able” and 100 indicating “as able as before”. The final CETI score represents the average across all items, with higher scores reflecting better communication abilities in daily life. The I-CETI is a not-time consuming, with a good reliable and administrable from expert clinicians and caregivers independently, with a high rate of agreement.

To evaluate depressive symptoms, we used the Aphasic Depression Rating Scale (ADRS) [27], which assesses depression in aphasic patients during the subacute phase of stroke. It includes items related to gastrointestinal symptoms, hypochondriasis, weight loss, apparent sadness, facial mimic, and insomnia. The scale is clinician-rated based on direct observation of patient behavior. Each item is scored from 0 (absent behavior) to 4 (severe presence). The total score ranges from 0 to 32, with scores ≥9 indicating significant depressive symptoms.

Functional autonomy in daily life was measured with the Activities of Daily Living (ADL) scale [28], which assesses independence in six basic self-care functions: bathing, dressing, toileting, transferring, continence, and feeding. Each function is scored as independent (yes) or dependent (no). A total score of 6 indicates full independence, 4 indicates moderate impairment, and 2 or less indicates severe functional impairment (score range: 0–6).

To evaluate the broader impact of emotional, cognitive, and physical impairments on daily functioning, we used the Instrumental Activities of Daily Living (IADL) scale [29]. This tool assesses eight higher-level abilities (e.g., using the telephone, managing medications, handling finances) and rates each item as independent (yes) or dependent (no). The total score ranges from 0 (low function) to 8 (high function), and the assessment takes approximately 10–15 min to complete.

Finally, the presence of dysphagia was assessed using the Dysphagia Outcome and Severity Scale (DOSS) [30], a 7-point scale used to evaluate the severity of swallowing difficulties and to guide dietary and nutritional recommendations. Scores range from 7 (normal swallowing function) to 1 (severe impairment).

## 3. Statistical Analysis

Differences in the distribution of categorical variables among groups were assessed using the chi-square test, whereas continuous variables were analyzed by means of nonparametric tests. Specifically, as a first step, we analyzed the demographical and clinical characteristics of the sample at baseline, and the prevalence and the characteristics of language impairments. Then, we verified patient’s clinical evolution by comparing clinical and language scores recorded at study entry and at discharge, by means of non-parametric tests for repeated measures (Wilcoxon Test, with alpha level set at *p* < 0.003, according to Bonferroni’s correction referred to 16 contrasts.) applied to each score recorded at T0 and T1.

Moreover, we compared clinical, demographical and linguistic variables between patients grouped as function of sex, etiology (hemorrhagic vs. ischemic) and dysphagia (present vs. absent), by means of non-parametric tests for independent samples (Mann–Whitney U test, with alpha level set at *p* < 0.003, according to Bonferroni’s correction referred to 16 contrast).

Lastly, the associations between clinical, demographical and language scores (predictors) with I-CETI delta score (∆CETI, dependent variable) (i.e., the difference between I-CETI at discharge and I-CETI at study entry) were assessed by means of two multivariable linear regression analyses with forward stepwise selection (*p* > 0.05 for exclusion). Age, sex, time post-injury, etiology, presence of tracheotomy, presence of dysphagia, were entered as control variables at Step 1 in both regressions. The predictors entered at Step 2 differed in the two analyses. In the first regression model, we assessed if clinical-demographical characteristic of patients (e.g., DOSS, ADL, IADL, ADRS) at study entry predict the recovery of functional communication abilities, as measured by means of ∆CETI. In the second, we explored the contribution of single language (e.g., BEL-II sub-scores and TOKEN scores) domains in the prediction of ∆CETI. Statistical analyses were computed by SPSS version 24.0 (SPSS, Chicago, IL, USA).

## 4. Results

The sample consisted of 61 patients with left ischemic or hemorrhagic stroke admitted in rehabilitation unit. Characteristics of patients at study entry are shown in Table 1.

Forty-six patients had suffered from brain injury less than 1 month before admission, 10 patients were admitted between 1- and 6-months post-onset and 5 were admitted more than 6 months after onset (range: 4–120 days post-onset). Most patients (54.5%) showed severe impairments (scores on BEL-II ≤ 30/100) on oral expression, 49.1 % had severe impairments in comprehension, and 64.9% had difficulties in repetition of words and sentences; writing was severely impaired in most patients (89.4%).

Most patients (60.2%) presented severe functional impairments in daily life activities as assessed by IADL, while 19.2% showed moderate functional impairments. Thirteen patients (19.1%) showed clinically relevant signs of depression at study entry.

At follow-up assessment, performed after a mean period of 91.2 days from study entry, 8 patients dropped out (5 for clinical complications; 3 for anticipated discharge). Complete follow-up data were therefore available for 53 patients.

Analysis of longitudinal data (Table 2) showed a significant global improvement of language abilities, as indicated by total scores of BEL-II, with an improvement in all language domains Moreover, a significant improvement was observed in functional independence activities of daily living measures and in depression.

Comparisons between clinical and linguistic scores showed several significant differences associated with presence of dysphagia: (i) ADL and IADL scores were significantly higher in non-dysphagic patients; (ii) the recovery of oral expression was higher in non-dysphagic patients. No significant differences were found between groups as function of etiology and sex (see Table A1, Table A2 and Table A3).

Finally, the results of the first hierarchical multiple regression (Step 1) showed that the ADL score was significantly associated with the recovery of functional communication abilities, as measured by the I-CETI (ADL score, β = 0.51; *p* < 0.001). The second regression model revealed that scores on the comprehension domain (Token score and Oral Comprehension on BEL-II) were also significantly associated with recovery (Token, β = 0.68; *p* < 0.05; BEL-II OC score, β = 0.75; *p* < 0.01). Descriptive statistics for each variable included in the regression analysis are presented in Table 1 and Table 2, grouped according to the selected predictors. The correlation matrix of all continuous variables included in the regression analysis is reported in Table A4.

## 5. Discussion

This study aimed to identify clinical and linguistic factors associated with the recovery of functional communication in patients with post-stroke aphasia, by means of validated tool (i.e., I-CETI) [19]. Our results primarily indicate that, although general improvements were observed across language domains in the entire sample—as shown by within-group analysis—functional communication outcomes were specifically predicted by comprehension abilities rather than by demographic or general clinical variables.

Functional communication deficits in aphasic patients often manifest as difficulties not only in producing and understanding language but also in effectively using language in everyday social interactions. These impairments can affect patients’ ability to convey needs, participate in conversations, and interpret contextual and non-verbal cues, leading to significant limitations in social participation and quality of life [4,5,9].

The comprehension is an important component of language skill and the comprehension deficits are very frequent in patients with aphasia. In language rehabilitation of patient with aphasia, the comprehension is considered a critical skill because it is predictive of cognitive, linguistic and social functioning [31]. Moreover, severe auditory comprehension deficits have an important negative influence on functional outcome and quality of life [32].

Specifically, as expected, language abilities measured by a traditional assessment tool for language impairments (i.e., BEL-II) [25], significantly improved over the course of rehabilitation, reflecting the potential for language recovery even during the subacute phase. Consistent with previous findings [3,33], these improvements were accompanied by gains in daily functional independence (ADL/IADL), while no significant reduction in depressive symptoms was observed [10,13]. Importantly, our data showed that language recovery was not uniformly distributed across domains—comprehension (both oral and written), oral expression and copying were most closely linked to functional communication gains. Among the factors related with the recovery of aphasia, most of the studies underline the importance of clinical and demographic factors such as age, gender, handedness, treatment, lesion size and location and aphasia initial severity [34]. El Hachioui et al. [35] showed that the phonological abilities are the most important linguistic predictor of post stroke aphasia outcome. More recently, Glize et al. [36] in a sample of 86 patients with severe aphasia found that word repetition task is a significant predictor of recovery, showing the importance of production in the language outcome.

On the other hand, with regard of functional communication recovery, few studies had investigated the influence of specific language skills on functional communication abilities [37]. In our study, we highlighted the role of oral comprehension at beginning of language rehabilitation treatments. In fact, our regression analyses revealed that comprehension abilities—specifically, performance on the Token Test [26] and the oral comprehension subtest of the BEL-II [25] —were the strongest predictors of functional communication recovery. This finding aligns with studies suggesting that understanding spoken language is critical for everyday interactions and may serve as a foundation for engaging in functional dialogue [8,22,37]. This result, emphasize the crucial role of receptive language in enabling patients to participate in communicative exchanges, especially in naturalistic settings, rather than expressions ability.

In line with previous studies [10,20], we found that the functional autonomy, as represented by basic activities in daily living (ADL), was associated with functional communication recovery after stroke. This result underlines the interdependence between communication abilities and overall functional autonomy. Patients with greater independence in basic self-care at baseline demonstrated better improvements in communication effectiveness. This suggests that rehabilitation programs aiming to improve aphasic patients’ autonomy should integrate comprehensive interventions addressing both language and daily functioning.

With regard to the possible contribution of clinical factors such as dysphagia on functional communication, interestingly, patients without dysphagia had better daily living skills at baseline and showed better functional outcomes, particularly in oral expression. Dysphagia may serve as a proxy indicator of more widespread neurological damage, including motor and cognitive domains, which could interfere with rehabilitation progress. Although dysphagia itself did not independently predict communication recovery in the regression models, its association with poorer initial performance and less improvement highlights the need for comprehensive management in these patients. In a recent study, Kaylor et al. [38] showed that in 38% of patients, aphasia, dysarthria and dysphagia had co-occurring diagnoses. Dysphagia was correlated with poor outcome. The multidisciplinary approach of post-stroke patients is an important requirement for adequate management of cognitive, linguistic, motor and clinical consequences of an acquired brain injury disease, such as stroke [39].

Moreover, in contrast with previous findings [40], demographic variables such as age, sex, and stroke etiology did not significantly influence communication recovery in our sample. However, this result should be interpreted with caution, as our sample was relatively homogeneous in terms of demographic characteristics, potentially limiting the variability necessary to detect such effects. Therefore, while our data support the stronger predictive value of initial comprehension abilities, they do not allow for definitive conclusions regarding the comparative influence of demographic factors. Future studies involving more demographically diverse populations are needed to better understand how these variables interact with linguistic and clinical predictors of functional recovery.

A key strength of our study is the use of an ecologically valid tool that incorporates caregiver perspectives on the patient’s ability to communicate in real-world contexts. While traditional measures often focus on structured language tasks, functional communication tools like the I-CETI [16] are essential for evaluating the true impact of aphasia on daily life. Our findings support the inclusion of such tools in routine assessments and in outcome measurement for rehabilitation programs. More research are need to confirm our results in order to improve the rehabilitation program of patients with aphasia, developing more tailored training.

### 5.1. Limitations

Several limitations should be acknowledged. First, the sample size, while adequate for our analyses, limits generalizability. Second, the study was conducted in specialized rehabilitation units, which may not reflect outcomes in less intensive settings. Third, while we assessed a range of linguistic domains, other cognitive variables such as attention and working memory—known to affect communication—were not directly measured. Lastly, caregiver ratings may be influenced by subjective perceptions, though this is also what gives the I-CETI ecological relevance.

### 5.2. Clinical Implications and Future Directions

These results highlight the importance of targeting comprehension skills in rehabilitation protocols, as gains in this domain are most closely associated with improved functional communication. Multidisciplinary approaches that integrate speech therapy, occupational therapy, and caregiver involvement are likely to be most effective. Future research should explore long-term outcomes beyond discharge and examine the predictive value of other cognitive and psychosocial variables. Additionally, incorporating neuroimaging data could help clarify the neural correlates of functional recovery.

## 6. Conclusions

Our study underscores the predictive value of language comprehension and functional autonomy in daily living on the early phase of post-stroke rehabilitation for functional communication in aphasic patients. These findings advocate for comprehensive, domain-specific assessments and support the implementation of individualized, function-oriented therapeutic strategies to optimize real-world communication outcomes.

## Figures and Tables

**Table 1:** Socio-demographical and clinical characteristics of patients at study entry.

		Patients = 61
Gender (n)	Female/Male	29/32
Mean age (±SD; years)		61.42 ± 14.1
Education (±SD; years)		8.9 ± 5.2
Stroke aetiology (n)	Ischemic/Hemorrhagic	39/22
Time post onset	Less than 1 month	46
	1–6 months	10
	More than 6 months	5
Mean disease duration (±SD; days)		28.2 ± 30.5
DOSS		4.28 ± 2.116

**Table 2:** Mean scores of clinical scales at study entry and at discharge.

Clinical Scales	Study Entry (Mean ± SD) N = 61	Discharge (Mean ± SD) N = 53	Paired *T*-Test	*p* Value
BEL-II Total score	40.64 ± 31.9	55.85 ± 33.5	3.700	**0.001**
Verbal expression	20.5 ± 33.2	26.3 ± 33.9	1.168	0.248
Oral comprehension	42.4 ± 39.5	70.4 ± 35.3	6.652	**<0.001**
Repetition	30.3 ± 40.1	48.6 ± 42.9	4.269	**<0.001**
Writing	10.1 ± 25.9	27.6 ± 40.2	3.903	**<0.001**
Reading comprehension	34.2 ± 44.3	52.1 ± 45.1	3.634	**0.001**
Reading	18.7 ± 34.1	36.5 ± 43.9	4.129	**<0.001**
Dictation	13.1 ± 30.1	35.3 ± 42.5	4.662	**<0.001**
Copy	30.4 ± 42.1	62.6 ± 45.1	5.383	**<0.001**
TOKEN	10.1 ± 10.9	16.1 ± 12.1	6.077	**<0.001**
ADRS	6.8 ± 4.4	7.21 ± 4.26	0.585	0.562
ADL	1.2 ± 2.1	2.3 ± 2.1	−3.015	0.004
IADL	0.56 ± 1.0	1.3 ± 1.8	−3.221	**0.002**
I-CETI	22.4 ± 27.2	52.3 ± 31.2	−8.221	**<0.001**

In bold are reported *p* value significantly equal or lower than alpha value set at 0.002 according to Bonferroni’s correction. BEL-II: Brief Exam of Language—II; ADRS: Aphasic Depression Rating Scale; ADL: Activity of Daily Living; IADL: Instrumental Activity of Daily Living; I-CETI: Italian version of the Communicative Effectiveness Index.

## Data Availability

The data presented in this study are available on request from the corresponding author due to privacy and ethical reasons.

## Abbreviations

The following abbreviations are used in this manuscript:

I-CETI	Italian version of Communicative Effectiveness Index questionnaire
BEL-II	Brief Exam of Language version II
ADL	Activities of Daily Life
IADL	Instrumental Activities of Daily Life
DOSS	Dysphagic Outcome and Severity Scale

## Appendix A

**Table A1:** Median scores and ranges of clinical and linguistic scales as function of sex.

Clinical Scales	Male Median (Min–Max) N = 28	Female Median (Min–Max) N = 25	U-Mann–Whitney	*p* Value
Age	63 (18–75)	67 (31–75)	310.5	0.481
Disease Duration (days)	17 (5–95)	18 (6–81)	313	0.509
Etiology (H/I)	9/19	7/18	0.108	0.743
Dysphagic (y/n)	17/11	17/8	0.305	0.581
DOSS	6 (1–7)	4 (1–7)	278.5	0.196
I-CETI	19.8 (0.1–93)	4.4 (0.1–70)	250	0.071
Token score	7 (0–34)	1.5 (0–30)	245	0.257
BEL II—Verbal expression	8.30 (0.1–96.6)	0.1 (0.1–80)	338	0.811
Oral comprehension	51.6 (0.1–100)	15 (0.1–100)	263	0.117
Repetition	18.3 (0.1–100)	0.1 (0.1–100)	284.5	0.206
Writing	0.1 (0.1–97.3)	0.1 (0.1–100)	310	0.296
Reading comprehension	25.0 (0.1–100)	0.1 (0.1–100)	291	0.259
Reading	0.1 (0.1–97)	0.1 (0.1–100)	289.5	0.185
Dictation	0.1 (0.1–100)	0.1 (0.1–100)	304	0.263
Copy	40.0 (0.1–100)	0.00 (100)	255	0.051
ADRS	6.8 (1–5)	7.2 (1–5)	0.585	0.562
ADL	1 (0–6)	0.1 (0–6)	263	0.081
IADL	0.1 (0–3)	0.1 (0–4)	313	0.395
∆CETI	30 (0.1–74.32)	25.2 (0.1–87)	338	0.830
∆BEL II -Verbal expression	0.1 (−13.3–86.6)	0.1 (−15–89.9)	297	0.235
∆BEL II—Oral expression	25 (−3.3–89.4)	11.6 (0.1–100)	347	0.957
∆BEL II—Repetition	6 (−15–95)	0.8 (−23.4–100)	327.5	0.679
∆BEL II—Writing	5 (−3.4–100)	0.1 (0.1–100)	226.5	0.012
∆BEL II—Reading comprehension	10 (−5.7–100)	0.1 (−2–95)	260.5	0.102
∆BEL II—Reading	8.40 (−23.3–100)	0.1 (−19.4–76.7)	200.5	0.05
∆BEL II—Dictation	1.60 (0.1–100)	0.1 (0.1–90)	258	0.073
∆BEL II—Copy	20.0 (−30–100)	0.1 (0–100)	347.5	0.62
∆ADL	1 (−5–6)	1 (−5–6)	340	0.854
∆IADL	1 (−2–6)	0.1 (−4–5)	311	0.461

DOSS: Dysphagia Outcome and Severity Scale; BEL-II: Brief Exam of Language—II; ADRS: Aphasic Depression Rating Scale; ADL: Activity of Daily Living; IADL: Instrumental Activity of Daily Living; I-CETI: Italian version of the Communicative Effectiveness Index. ∆: means the scores at T1 minus scores at T0.

**Table A2:** Median scores and ranges of clinical and linguistic scales as function of etiology.

Clinical Scales	Haemorrhagic Median (Min–Max) N = 18	Ischemic Median (Min–Max) N = 35	U-Mann–Whitney	*p* Value
Age	63 (35–75)	65 (18–75)	197.5	0.56
Disease Duration	26.5 (8–81)	15 (5–95)	158.0	0.007
Sex (m/f)	9/7	19/18	0.108	0.743
Dysphagic (y/n)	10/6	24/13	0.027	0.869
DOSS	4 (1–7)	5.0 (1–7)	268	0.582
Token score	4 (0–28)	5 (0–34)	2.57.5	0.886
I-CETI	48 (0.1–100)	55.3 (0.1–100)	224.5	0.161
BELII—Verbal expression	33.3 (0.1–75)	0.1 (0.1–96.6)	257	0.398
BELII—Oral comprehension	20 (0.1–100)	30 (0.1–100)	279.5	0.747
BELII—Repetition	5 (0.1–100)	0.1 (0.1–100)	290	0.900
BELII—Writing	0.1 (0.1–60)	0.1 (0.1–100)	265	0.379
BELII—Reading comprehension	0.1 (0.1–100)	3 (0.1–100)	271.5	0.610
BELII—Reading	0.1 (0.1–96.6)	0.1 (0.1–100)	295	0.981
BELII—Dictation	0.1 (0.1–100)	0.1 (0.1–100)	287	0.812
BELII—Copy	40.1 (0.1–100)	68 (0.1–98)	250	0.304
ADRS	6.3 (1–5)	7.1 (1–5)	0.547	0.592
ADL	0.1 (0–6)	1 (0–6)	223	0.111
IADL	0.1 (0–3)	0.1 (0–4)	272.5	0.557
∆CETI	30 (0.1–68)	25.3 (0.1–87.06)	258.5	0.467
∆BEL II—Verbal expression	0.1 (0.1–86.6)	0.1 (−15–89.9)	290	0.884
∆BEL II—Oral comprehension	35 (0.1–85)	11.6 (−3.3–100)	233	0.217
∆BEL II—Repetition	6.6 (−16.7–95)	0.1 (−23.4–100)	256	0.424
∆BEL II—Writing	0.1 (0.1–100)	0.1 (−3.4–100)	264	0.477
∆BEL II—Reading comprehension	7.5 (0.1–100)	3.4 (−5.70–100)	282.5	0.789
∆BEL II—Reading	1.6 (−10–100)	0.1 (−23.33–96.6)	286	0.893
∆BEL II—Dictation	0.1 (0.1–100)	0.1 (0.1–100)	274	0.641
∆BEL II—Copy	60 (0.1–100)	0.1 (−30–100)	196.5	0.039
∆ADL	1 (0–5)	1 (−5–6)	221	0.134
∆IADL	1 (0–5)	0.1 (−4–6)	233	0.195

DOSS: Dysphagia Outcome and Severity Scale; BEL-II: Brief Exam of Language—II; ADRS: Aphasic Depression Rating Scale; ADL: Activity of Daily Living; IADL: Instrumental Activity of Daily Living; I-CETI: Italian version of the Communicative Effectiveness Index. ∆: means the scores at T1 minus scores at T0.

**Table A3:** Median scores and ranges of clinical and linguistic scales as function of presence of dysphagia.

Clinical Scales	Dysphagic Median (Min–Max) N = 34	Non-Dysphagic Median (Min-Max) N= 19	U-Mann–Whitney	*p* Value
Age	66 (36–75)	56 (18–75)	182.0	0.009
Sex (m/f)	17/17	11/8	0.305	0.581
Disease Duration	16.5 (5–95)	20 (6–81)	315.5	0.889
Etiology (H/I)	10/24	6/13	0.027	0.869
DOSS	3 (1–6)	6 (5–7)	15.5	**0.001**
Token	0.1 (0.1–34)	8 (0.1–30)	159.5	0.015
I-CETI	50 (0.1–96)	70 (10–100)	199.5	0.02
BELII–Verbal expression	0.1 (0.1—96.6)	9 (0.1–80)	238.0	0.078
BELII—Oral comprehension	15 (0.1–100)	65 (0.1–100)	187.5	0.011
BELII—Repetition	0.1 (0.1–100)	60 (0.1–100)	191.0	0.008
BELII—Writing	0.1 (0.1–97.3)	0.1 (0.1–100)	235.5	0.017
BELII—Reading comprehension	0.1 (0.1–100)	43.3 (0.1–100)	217.0	0.035
BELII—Reading	0.1 (0.1–97)	16.6 (0.1–100)	201.5	0.006
BELII—Dictation	0.1 (0.1–100)	0.1 (0–100)	231.0	0.02
BELII—Copy	0.1 (0.1–100)	50 (0.1–100)	196.5	0.007
ADRS	6.4 (1–5)	6.9 (1–5)	0.524	0.602
ADL	0.1 (0–6)	1 (0–6)	171.5	**0.002**
IADL	0.1 (0–2)	1 (0–4)	186.0	**0.001**
∆CETI	30 (0.1–87.06)	28 (0.93–74.3)	314.5	0.875
∆BEL II—Verbal expression	0.1 (−3.3–89.40)	0.1 (0.1–89.9)	190.5	**0.002**
∆BEL II—Oral expression	18.4 (−80–89.4)	10 (0–100)	319.0	0.940
∆BEL II—Repetition	0.1 (−23.4–91.6)	22.7 (−1.7 −100)	215.0	0.039
∆BEL II–Writing	0.1 (−3.4–100)	13.3 (0.1–100)	182.0	0.003
∆BEL II—Reading comprehension	0.1 (−5.7–100)	5 (−2–100)	233.0	0.087
∆BEL II—Reading	0.1 (−10.7–90)	11.6 (−23–100)	203.0	0.019
∆BEL II—Dictation	0.1 (0.1–100)	20 (0.1–100)	229.0	0.056
∆BEL II—Copy	0.1 (−30–100)	30 (−20–100)	262.5	0.230
∆ADL	1 (−5–6)	1 (−5–5)	289.0	0.515
∆IADL	0.1 (−2–4)	0.1 (−4–6)	321.5	0.976

In bold are reported *p* value significantly equal or lower than alpha value set at 0.002 according to Bonferroni’s correction. DOSS: Dysphagia Outcome and Severity Scale; BEL-II: Brief Exam of Language—II; ADRS: Aphasic Depression Rating Scale; ADL: Activity of Daily Living; IADL: Instrumental Activity of Daily Living; I-CETI: Italian version of the Communicative Effectiveness Index. ∆: means the scores at T1 minus scores at T0.

**Table A4:** Correlations between measure of clinical, functional and language scales.

	1	2	3	4	5	6	7	8	9	10	11	12	13	14	15	16	17
1. Age	1																
2. Disease duration	−0.311 *	1															
3. Token score	−0.085	−0.186	1														
4. DOSS	−0.369 **	−0.173	0.453 **	1													
5. ADL	−0.139	−0.195	0.489 **	0.546 **	1												
6. IADL	−0.094	−0.082	0.573 **	0.494 **	0.783 **	1											
7. BEL-II VE	−0.026	−0.039	0.692 **	0.433 **	0.427 **	0.678 **	1										
8. BEL-II OC	−0.140	−0.099	0.820 **	0.400 **	0.525 **	0.545 **	0.605 **	1									
9. BEL-II R	−0.087	−0.087	0.390 **	0.424 **	0.385 **	0.442 **	0.726 **	0.391 **	1								
10. BEL-II W	−0.030	−0.117	0.650 **	0.294 *	0.395 **	0.546 **	0.777 **	0.499 **	0.348 **	1							
11. BEL-II RC	−0.122	−0.199	0.761 **	0.406 **	0.593 **	0.651 **	0.729 **	0.828 **	0.398 **	0.630 **	1						
12. BEL-II R	−0.108	−0.005	0.596 **	0.413 **	0.415 **	0.567 **	0.767 **	0.542 **	0.780 **	0.577 **	0.625 **	1					
13. BEL-II D	−0.012	−0.204	0.681 **	0.323 **	0.418 **	0.577 **	0.610 **	0.511 **	0.413 **	0.787 **	0.610 **	0.532 **	1				
14. BEL-II C	−0.184	−0.211	0.715 **	0.396 **	0.559 **	0.636 **	0.643 **	0.661 **	0.304 *	0.744 **	0.834 **	0.596 **	0.681 **	1			
15. ADRS	−0.199	−0.145	0.258	0.228	−0.188	0.409 *	0.106	0.126	0.147	0.062	0.380	0.558 **	0.072	0.064	1		
16. I-CETI	−0.066	−0.300 *	0.737 **	0.453 **	0.672 **	0.667 **	0.613 **	0.793 **	0.371 **	0.523 **	0.765 **	0.521 **	0.509 **	0.670 **	0.314 *	1	
17. ∆CETI	−0.164	−0.328 *	0.524 **	0.364 **	0.496 **	0.490 **	0.473 **	0.604 **	0.367 **	0.325 *	0.584 **	0.409 **	0.449 **	0.475 **	0.127	0.652 **	1

DOSS: Dysphagia Outcome and Severity Scale; BEL-II: Brief Exam of Language—II; ADRS: Aphasic Depression Rating Scale; ADL: Activity of Daily Living; IADL: Instrumental Activity of Daily Living; I-CETI: Italian version of the Communicative Effectiveness Index. ∆: means the scores at T1 minus scores at T0. Spearman’s coefficients: * asterisk indicates *p* value < 0.05; ** indicates *p* value < 0.01.
